# The prognostic impact of abdominal surgery in cancer patients with neutropenic enterocolitis: a systematic review and meta-analysis, on behalf the Groupe de Recherche en Réanimation Respiratoire du patient d’Onco-Hématologie (GRRR-OH)

**DOI:** 10.1186/s13613-018-0394-6

**Published:** 2018-04-19

**Authors:** Colombe Saillard, Lara Zafrani, Michael Darmon, Magali Bisbal, Laurent Chow-Chine, Antoine Sannini, Jean-Paul Brun, Jacques Ewald, Olivier Turrini, Marion Faucher, Elie Azoulay, Djamel Mokart

**Affiliations:** 1 Haematology Department, Institut Paoli Calmettes, 232 Boulevard Sainte Marguerite, 13009 Marseille Cedex 09, France; 2 Medical Intensive Care Unit, Saint-Louis University Hospital, AP-HP, Paris, France; 3 Medical-Surgical Intensive Care Unit, Hôpital Nord, Université Jean Monnet, Saint Etienne, France; 4 GRRR-OH (Groupe de Recherche en Réanimation Respiratoire du patient d’Onco-Hématologie), Paris, France; 5 Polyvalent Intensive Care Unit, Department of Anesthesiology and Critical Care, Institut Paoli Calmettes, Marseille, France; 6 Surgery Department, Institut Paoli Calmettes, Marseille, France; 7 Faculté de Médecine, Université Paris Diderot, Sorbonne-Paris-Cité, Paris, France

**Keywords:** Neutropenic enterocolitis, Typhlitis, Cancer patients, Abdominal surgery, Meta-analysis

## Abstract

Neutropenic enterocolitis (NE) is a diagnostic and therapeutic challenge associated with high mortality rates, with controversial opinions on its optimal management. Physicians are usually reluctant to select surgery as the first-choice treatment, concerns being raised regarding the potential risks associated with abdominal surgery during neutropenia. Nevertheless, no published studies comforted this idea, literature is scarce and surgery has never been compared to medical treatment. This review and meta-analysis aimed to determine the prognostic impact of abdominal surgery on outcome of neutropenic cancer patients presenting with NE, versus medical conservative treatment. This meta-analysis included studies analyzing cancer patients presenting with NE, treated with surgical or medical treatment, searched by PubMed and Cochrane databases (1983–2016), according to PRISMA recommendations. The endpoint was hospital mortality. Fixed-effects models were used. The meta-analysis included 20 studies (385 patients). Overall estimated mortality was 42.2% (95% CI = 40.2–44.2). Abdominal surgery was associated with a favorable outcome with an OR of 0.41 (95% CI = 0.23–0.74; *p* = 0.003). Pre-defined subgroups analysis showed that neither period of admission, underlying malignancy nor neutropenia during the surgical procedure, influenced this result. Surgery was not associated with an excess risk of mortality compared to medical treatment. Defining the optimal indications of surgical treatment is needed.

*Trial registration* PROSPERO CRD42016048952

## Background

Neutropenic enterocolitis (NE) or typhlitis is a serious complication of neutropenia characterized by segmental ulceration and inflammation with necrosis of ileum, cecum and ascending colon [1]. NE was initially described in an autopsy study of children with acute leukemia [2] and evolved to an entity encountered in neutropenic patients [3–8]. The pathogenesis of NE is poorly understood and probably multifactorial. Immunosuppression induced by neutropenia, combined with chemotherapy toxicity, tumoral infiltration, intramural hemorrhage and inflammatory reaction lead to direct mucosal injury, up to necrotizing damages and microbial translocation. Patients typically present with gastrointestinal (GI) symptoms, in a context of neutropenia, usually following chemotherapy, with bowel wall thickening and positive microbiological samples. Recently, revised diagnostic criteria have been proposed [9]. NE incidence is unknown, reports ranging from 0.8 to 26% [8]. NE carries a poor prognosis, with mortality rates up to 80%, due to complications such as bowel perforation, ischemia, necrosis and septic shock evolution [5, 9, 10].

NE optimal management is controversial, with some advising abdominal surgery [4, 11–16], and others advocating medical conservative treatment including broad-spectrum antibiotherapy, bowel rest and general supportive care [8, 17, 18]. Physicians are often reluctant to surgery, because of neutropenia and thrombopenia. When surgery is indicated, the question of delaying it until neutropenia resolution arises.

Major advances have been made in the last decade in onco-hematology patients, particularly in the management of septic shock [19, 20], critically ill onco-hematology patients admitted to the intensive care unit (ICU) [21], neutropenic cancer patients [12, 22] and organ failures including acute respiratory failure [23–27]. Surprisingly, no major improvements have been reported in neutropenic cancer patients presenting with surgical acute abdominal syndrome [28]. Surgical treatment has never been evaluated neither compared to medical treatment, NE being rare, literature scarce and mainly based on small observational reports, case series or case reports. Surgeons and onco-hematologists are usually reluctant to select surgery as the first-choice treatment, concerns being raised regarding the potential risks associated with abdominal surgery during neutropenia, which is furthermore frequently associated with thrombopenia. Nevertheless, no published studies comforted this idea. Moreover, neutropenia is not considered anymore as an unfavorable prognostic factor in critically ill cancer patients, as recently published in a large meta-analysis [22]. Surgery even appeared to be associated with a good prognosis in a recent publication in neutropenic cancer patients with acute abdominal pain [12].

To determine the prognostic impact of abdominal surgery, compared to medical conservative treatment, on short-term mortality of neutropenic cancer patients presenting with NE, we conducted a systematic review and meta-analysis. Secondary objectives were to assess the influence of surgery on outcome in pre-specified subgroups, according to underlying malignancy, period of admission and the presence of neutropenia during the surgery procedure.

## Methods

### Review

These systematic review and meta-analysis were reported following criteria set by the PRISMA (Preferred Reporting Items for Systematic reviews and Meta-Analyses) statement and the MOOSE (Meta-analysis Of Observational Studies in Epidemiology) group [29–34]. This study was registered on the international register for prospective reviews PROSPERO (number CRD42016048952).

### Study outcome

The aim of this meta-analysis was to determine the prognostic impact of abdominal surgery, compared to medical treatment, on short-term outcome of neutropenic cancer patients presenting with NE. The selected endpoint was overall hospital mortality.

### Search strategy and eligibility assessment

First, public-domain databases including PubMed and the Cochrane database were searched by using exploded Medical Subject Headings and the appropriate corresponding keywords: “NEUTROPENIC ENTEROCOLITIS” OR “TYPHLITIS.” The research was restricted to English-written abstracts with full-text articles available concerning humans from January 1983 to 2016. References cited in the articles of interest and published reviews were manually searched to find any additional reports. The search was rerun immediately prior to analysis to ensure that the most current information was presented. Abstracts were carefully checked and studies focusing on children or patients aged lower than 18 years old, case reports and studies failing to focus on neutropenic patients were excluded. There were no restrictions in terms of underlying malignancy or study type. In case series, a minimum of three patients were needed with at least one patient in each treatment arm to be analyzed.

**Fig. 1:**
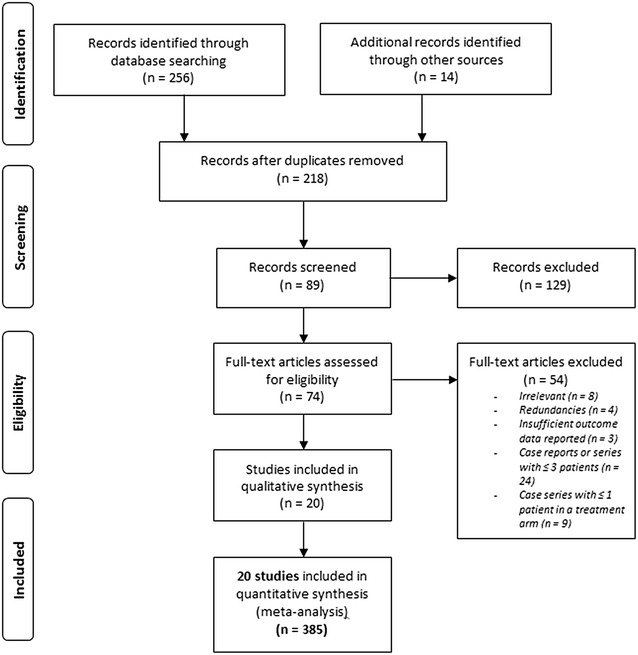
Flowchart of selected studies, according to PRISMA recommendations

All remaining references were then downloaded for consolidation, elimination of duplicates and further analysis. Four authors (CS, LZ, MD, DM) independently determined the eligibility of all studies identified in the initial research. Any disagreements were resolved by discussion. The flowchart of publications selection is presented in Fig. 1.

### Data extraction and quality assessment

The authors carried out data extraction working in pairs. Disagreements were resolved by discussion among authors and in case of persistent disagreement by adjudication of a third evaluator.

For each included trial, information was extracted on the following: study design, follow-up period, studied population, number of patients included, period of inclusion, median age, underlying malignancy, rate of allogeneic hematopoietic stem cell transplantation recipients, neutropenia duration, number of patients undergoing surgery during the neutropenic phase, outcome (overall hospital mortality) of patients with and without abdominal surgery, type of surgery, pathological findings and microbiological documentation.

Risk of bias was assessed using the Cochrane’s Tool to Assess Risk of Bias in Cohort Studies (http://methods.cochrane.org/bias/reporting-biases). However, all the studies were homogenous in terms of methodology, all of them being retrospective, single-center with small-sample size cohorts including many case series and case reports, making standard scale or checklists difficult to apply.

### Statistical analysis

Results were analyzed using Review Manager 5.1 (Cochrane Collaboration, Oxford, UK). Overall hospital mortality of included patients and mortality in included studies are reported as median (interquartiles). The summary estimates of odd ratios (OR) were calculated using the fixed-effects model and presented as forest plots after pooling. All estimates are presented as proportion with two-sided 95% confidence interval (95% CI). The pooled OR, symbolized by a solid diamond at the bottom of the forest plot (the width of which represents the 95% CI), is the best estimate of the pooled outcome. Publication bias was assessed by visually inspecting the funnel plot.

Three subgroups analyzes were preplanned, in order to evaluate the impact of abdominal surgery on outcome according to underlying malignancy (solid tumor, hematological malignancy or both), median ICU admission period (before or after 2003) and neutropenia the day of surgery defined by a neutrophil count < 0.5 G/L (when neutropenia status during surgery procedure was not specified, patients were not analyzed in this subgroup analysis).

A *p* value of less than 0.05 was considered statistically significant. Cochrane’s *χ*^2^ test and *I*^2^ test for heterogeneity were used to assess interstudy heterogeneity. The *χ*^2^ test assessed whether observed differences in results were compatible with chance alone, and the *I*^2^ described the percentage of the variability in effect estimates resulting from heterogeneity rather than from sampling error. An *I*^2^ test for heterogeneity above 0.25 was considered as moderate heterogeneity. Statistically significant heterogeneity was considered present at *χ*^2^ *p* < 0.10 and *I*^2^ > 50%. We used the fixed-effects model as heterogeneity was low in our analyses.

## Results

The initial search yielded 270 citations, of which 52 were excluded for duplication. Among these records, 129 were excluded as irrelevant to the scope of this review. For the 89 remaining records, abstracts were carefully checked, and 74 full-text articles focusing on NE cancer patients’ management were selected for further evaluation. Articles considered as irrelevant, redundant, with insufficient outcome data reported or less than three patients (including at least one in each treatment arm), or including patients under 18 were excluded. Finally, 20 studies, with a total of 385 patients fulfilled our eligibility criteria and were included (Fig. 1) [9–11, 35–50].

### Characteristics of included studies

Included studies were published from 1983 to 2015. All were retrospective and single-center, except one which included eight academic institutions [9]. Study designs consisted of small-size observational studies, case reports (including ≥ 3 patients) and cases series. The sample size of included patients ranged from 3 to 88 patients. Study populations varied across studies, including ten studies focusing on hematology patients (*n* = 229) [36–38, 40, 41, 43, 45, 47, 50], one on patients with solid malignancies (*n* = 4) [49] and the nine others on onco-hematology patients with no further details [9–11, 35, 39, 42, 44, 46, 48]. Allogenic hematopoietic stem cell recipients represented 93 patients (24%). The outcome variable was overall hospital mortality in all studies. On the total of 385 patients, 76 underwent abdominal surgery, versus 309 benefiting from medical conservative treatment. The detail of surgery procedures, pathological findings and microbial documentation is reported in Tables 1 and 2.

**Table 1:** Surgical procedures in patients undergoing abdominal surgery and pathological findings

Study and year of publication	*n*/*n*′	Surgical procedures	Pathological findings	Surgery indication	Mortality after surgery
Mulholland 1983	3/4	Right hemicolectomy with ileostomy (*n* = 1) Subtotal colectomy with ileostomy (*n* = 1) No resection (*n* = 1)	Extensive mucosal and submucosal necrosis. No perforation. The submucosa was edematous	Cecal perforation (2) Colic perforation (1)	2/3
Mower 1986	8/13	Laparotomy without resection (*n* = 3) Right hemicolectomy (*n* = 4) Terminal ileal resection (*n* = 1)	Isolated ileocecal inflammation, edema or pneumatosis without evidence of necrosis or infarction	Perforation (5) Exploratory laparotomy (3)	1/8 5/8 dead at 4 months
Moir 1986	6/16	Right hemicolectomy, ileostomy, and mucous fistula (*n* = 3) Divided ileostomy (*n* = 1) Local resection, ileostomy, abscess drainage (*n* = 2)	In all, cecal ulceration and mucosal thickening with intense submucosal and mucosal edema. The more severe cases showed hemorrhagic infarction	Severe systemic sepsis Persistent tenderness, rebound Pneumatosis Bowel instruction Paracolic abcesses Right flank myonecrosis	2/6
Starnes 1986	5/23	/	/	Cholecystitis Right lower quadrant typhlitis Splenic infarction Diverticular perforation Large bowel obstruction	0/5
Villar 1987	18/19	Exploratory laparotomy (*n* = 4) Colectomy (*n* = 3) Drainage of hepatic abscesses (*n* = 3) Enterolysis (*n* = 3) Simoid resection (*n* = 1) Cholecystectomy (*n* = 1) Appendicectomy (*n* = 1) Ligation of esophageal varices and splenectomy (*n* = 1) Meckel’s resection (*n* = 1)	/	Enterocolitis (4) Sepsis (3) Hepatic abscesses (3) Bowel obstruction (3) Cholecystitis (1) Appendicitis (1)	3/18
Wade 1992	6/22	Appendectomy (*n* = 2) No bowel resection (*n* = 1) Right hemicolectomy (*n* = 1) Diverting colostomy (*n* = 1) Cholecystectomy (*n* = 1)	/	Appendicitis (2) Digestive hemorrhage (1) Cecum perforation (1) Prevention of perineal excoriation (1) Cholecystitis (1)	3/6
Abbasoglu 1993	2/3	Appendectomy and cecum exteriorization (*n* = 1) Laparotomy and sigmoid exteriorization (*n* = 1)	Ulceration, thrombosed vessels and necrotic areas in the mucosa and submucosa. Necrosis involving all layers, sigmoid perforation	Appendix perforation Colon perforation	1/2
Buyukasik 1997	3/20	Bowel resection and enterostomy (*n* = 3)	Ischemic and hemorrhagic mucosal and submucosal necroses extending focally to serosal surface, microvascular thromboses, submucosal edema, bacterial infiltrates with the absence of inflammatory response and necrotic mucosal pseudo-membranes	/	0/3
Gomez 1998	1/18	Exploratory laparotomy (*n* = 1)	Edematous and thickened cecum and ascending colon	/	/
Song 1998	2/14	End jejunostomy and fistula (*n* = 1) No bowel resection (*n* = 1)	Ischemia of the entire small bowel, and right colon most severely involving the distal ileum with focal areas of transmural necrosis. Thickened inflamed cecum	Medical treatment failure (1) Peritoneal signs (1)	1/2
Ibrahim 2000	3/6	Right hemicolectomy (*n* = 1) Left hemicolectomy (*n* = 1) Left hemicolectomy (*n* = 1)	Necrotic bowel. Multiple ulcerations of the sigmoid colonic wall and acute and chronic inflammation, acute serositis and perforation. Histologically, extensive ischemic damage was evident, as well as vascular changes, including thrombosis and revascularization associated with mucosal regeneration Perforation of the descending sigmoid colon. Transmural necrosis associated with perforation was histologically evident	Pneumoperitoneum (2) Severe abdominal pain (1)	1/3
Cartoni 2001	1/88	Left hemicolectomy (*n* = 1)	Ulceration and hemorrhagic necrosis of the intestinal mucosa in all cases, together with a mild-to-moderate mononuclear inflammatory infiltrate	/	0/1
Gorschluter 2002	5/13	Cholecystectomy (*n* = 2) Left-sided colostomy (*n* = 1) Laparotomy (*n* = 1) Appendectomy (*n* = 1)	Diffuse serous inflammation	/	0/5
Kirkpatrick 2003	1/11	Total colectomy (*n* = 1)	Digestive perforation	/	/
Hsu 2004	2/9	Laparotomy (*n* = 2)	Bowel necrosis and peritonitis	/	/
Batlle 2007	6/7	Ileocolic resection (*n* = 1) Right hemicolectomy with ileostomy (*n* = 5)	Typhlitis confirmed. Ulcerated mucosa. Massive edema	/	/
Badgwell 2008	3/17	Right colectomy (*n* = 2) Left colectomy (*n* = 1)	/	/	0/3
Gondal 2010	4/16	/	/	/	/
Mokart 2017	58/58	/	/	No cause (1) Primary peritonitis (2) Tumoral infiltration (12) Digestive graft versus host disease (2) NE (3) Invasive digestive aspergillosis (3) Digestive bleeding (5) Appendicitis (2) Cholecystitis (3) Sigmoiditis (8) Gastrointestinal obstruction (8) Mesenteric ischemia (2) Others (7)	18/58
Sachak 2015	15/19	Segmental resection (*n* = 15)	Gross mucosal abnormalities with a patchy distribution. Histologic abnormalities always involved the cecum and/or right colon with other bowel segments variably involved. NE lesions were not seen in the appendix or rectum. Pathologic features included necrosis and hemorrhage. Many cases were characterized by infiltrating organisms in an inflammatory depleted background	/	4/19

*n* patients undergoing surgery, *n*′ total number of patients. *NE* neutropenic enterocolitis

**Table 2:** Microbial documentation reported in the selected studies

Type of samples	Pathogens identified
Blood cultures	Bacteria
	*Klebsiella pneumonia* (*n* = 2)
	*Pseudomonas aeruginosa* (*n* = 1)
	*Escherichia coli* (*n* = 14)
	*Enterococcus faecium* (*n* = 6)
	*Enterobacter aerogenes* (*n* = 1)
	*Clostridium septicum* (*n* = 1)
	*Aeromonas hydrophilia* (*n* = 1)
	*Clostridium perfringens* (*n* = 1)
	*Bacteroides fragilis* (*n* = 1)
	Gram-negative bacilli (non-specified) (*n* = 39)
	*Stenotrophomonas maltophilia* (*n* = 1)
	*Staphylococcus aureus* (*n* = 1)
	*Staphylococcus epidermidis* (*n* = 2)
	Alpha-hemolytic streptococcus (*n* = 1)
	Viridans streptococcus (*n* = 1)
	Gram-positive Cocci (non-specified) (*n* = 8)
	Bacteria (non-specified) (*n* = 13)
	Fungi
	*Candida krusei* (*n* = 1)
	*Candida glabrata* (*n* = 1)
	Fungemia (*n* = 4)
	Candida (non-specified) (*n* = 1)
	Virus
	Cytomegalovirus (*n* = 1)
Peroperative digestive samples	Bacteria
	*Pseudomonas aeruginosa* (*n* = 4)
	*Escherichia coli* (*n* = 1)
	*Klebsiella pneumonia* (*n* = 1)
	Diphteroides (*n* = 1)
	*Acinetobacter anitratus* (*n* = 1)
	*Clostridium difficile* (*n* = 2)
	*Bacteroides fragilis* (*n* = 1)
	*Enterobacter aerogenes* (*n* = 1)
	Gram-negative bacilli (non-specified) (*n* = 21)
	Gram-positive bacilli (non-specified) (*n* = 2)
	Fungi
	*Aspergillus fumigatus* (*n* = 1)
	*Candida glabrata* (*n* = 2)
	*Candida krusei* (*n* = 1)
	Candida (non-specified) (*n* = 7)
	*Virus*
	Cytomegalovirus (*n* = 1)
Autopsy samples	*Candida albicans* (*n* = 3)
	*Candida glabrata* (*n* = 1)
	*Aspergillus fumigatus* (*n* = 1)
	*Aspergillosis pneumonia* (*n* = 5)
	*Fungal pneumonia* (*n* = 3)
	Kidney and thyroid candida abscess (*n* = 1)
Stool samples	*Clostridium difficile* (*n* = 8)
	*Pseudomonas aeruginosa* (*n* = 1)
	*Escherichia coli* (*n* = 1)
	*Candida glabrata* (*n* = 2)
	Yeasts (non-specified) (*n* = 3)
	Adenovirus (*n* = 1)

### Outcome

**Fig. 2:**
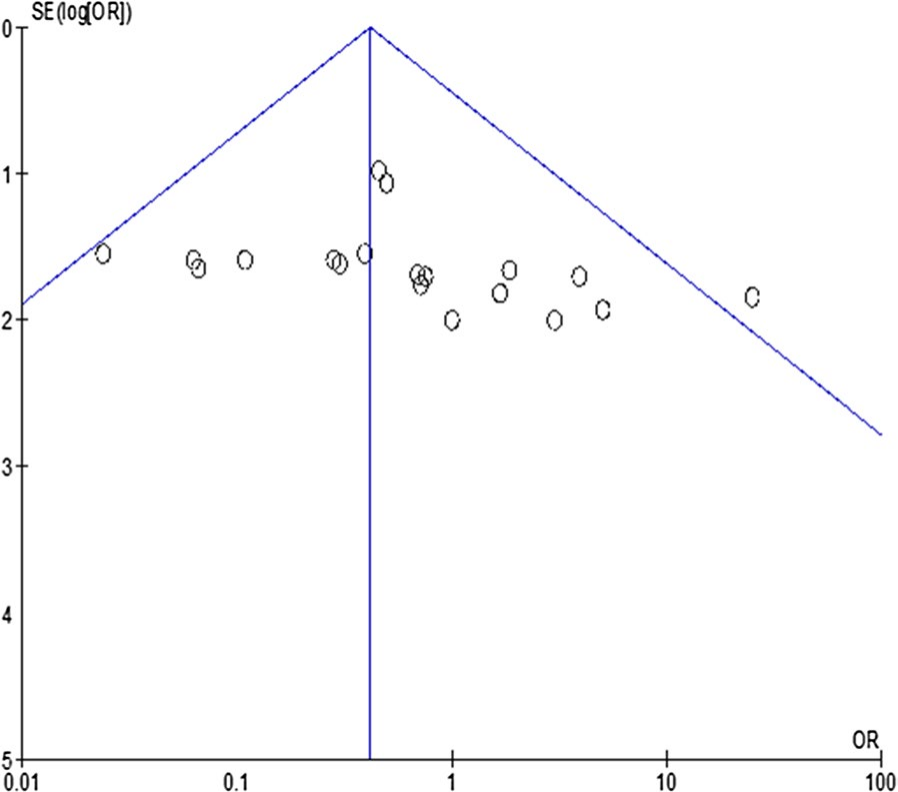
Funnel plot of included studies

**Fig. 3:**
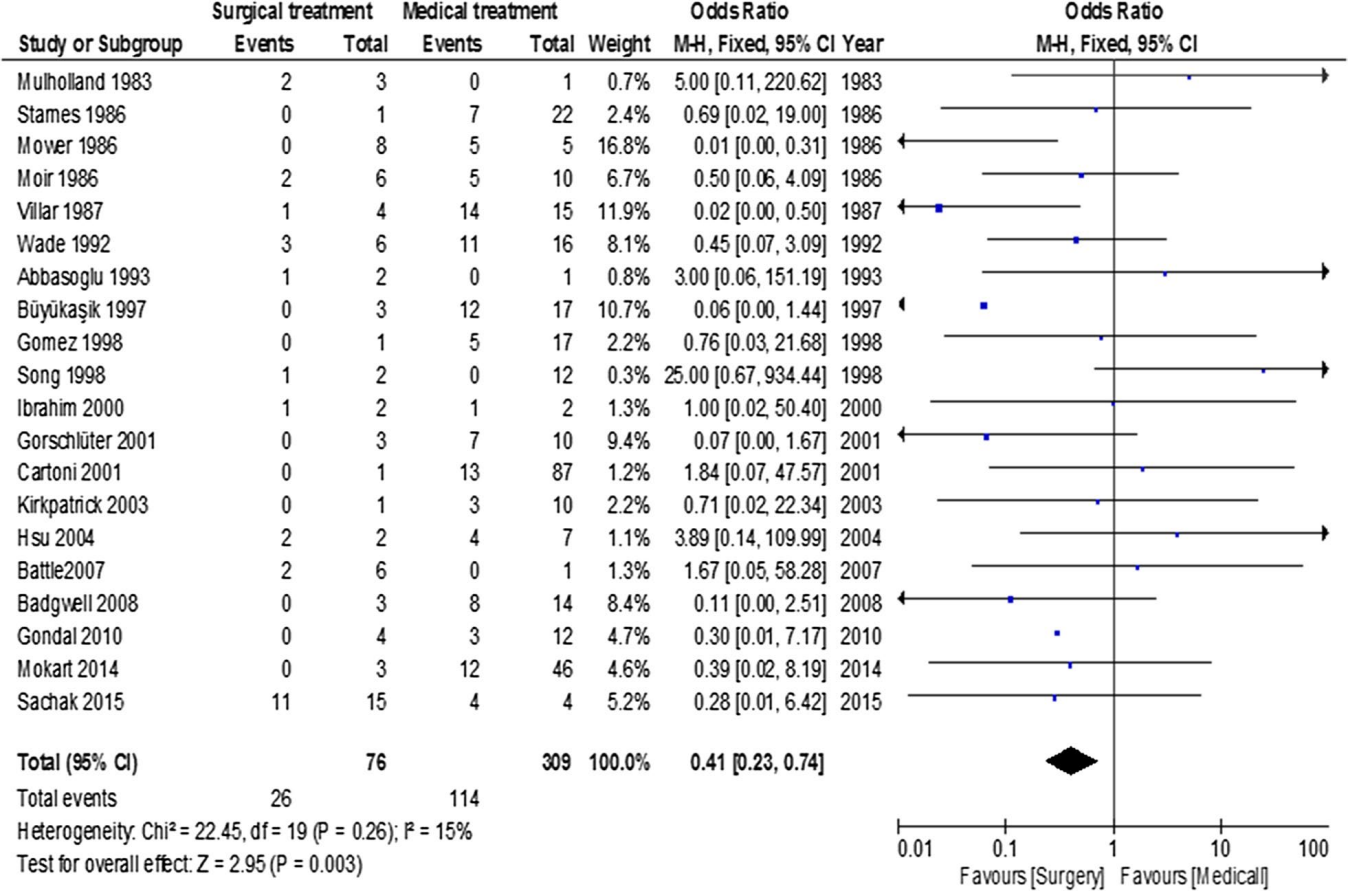
Summary of odds ratio in included studies according to treatment arm (abdominal surgery versus medical conservative treatment)

Overall estimated mortality rate was 42.2% (95% CI = 40.2–44.2). Overall estimated mortality rates of patients undergoing surgical or medical treatment were 26.6% (95% CI = 19.7–33.4%) and 43.7% (95% CI = 40.1–47.3%), respectively. Funnel plot analysis failed to identify publication bias (Fig. 2). Overall, abdominal surgery was not deleterious and was associated with a favorable outcome, compared to medical conservative treatment, with an OR of 0.41 (95% CI = 0.23–0.74; *p* = 0.003) (Fig. 3). Heterogeneity was low (*I*^2^ = 15%).

### Association of abdominal surgery with outcome in the pre-defined subgroups

- Influence of inclusion period (before or after 2003)

**Fig. 4:**
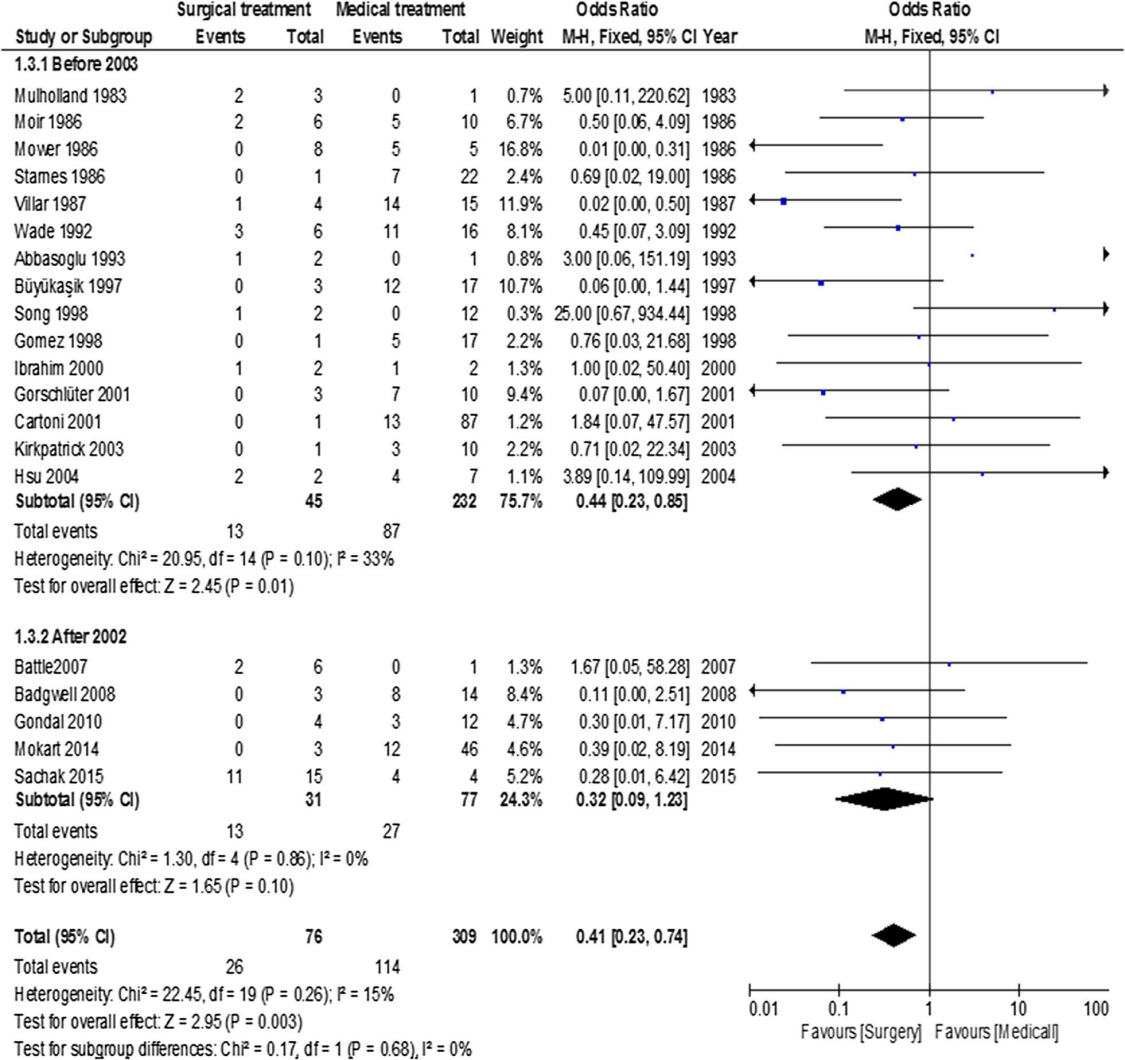
Summary of odds ratio in included studies according to inclusion period

Mortality according to the inclusion period is displayed in Fig. 4. Inclusion period did not modify the results of abdominal surgery in neutropenic cancer patients with NE. Before 2003, patients undergoing surgery had a better prognosis compared to patients receiving medical treatment, with an OR of 0.44 (95% CI = 0.23–0.85; *p* = 0.01). After 2003, the association of surgery with outcome tended to decrease over time, with an OR of 0.32 (95% CI = 0.09–1.23; *p* = 0.1).

- Influence of underlying malignancy

**Fig. 5:**
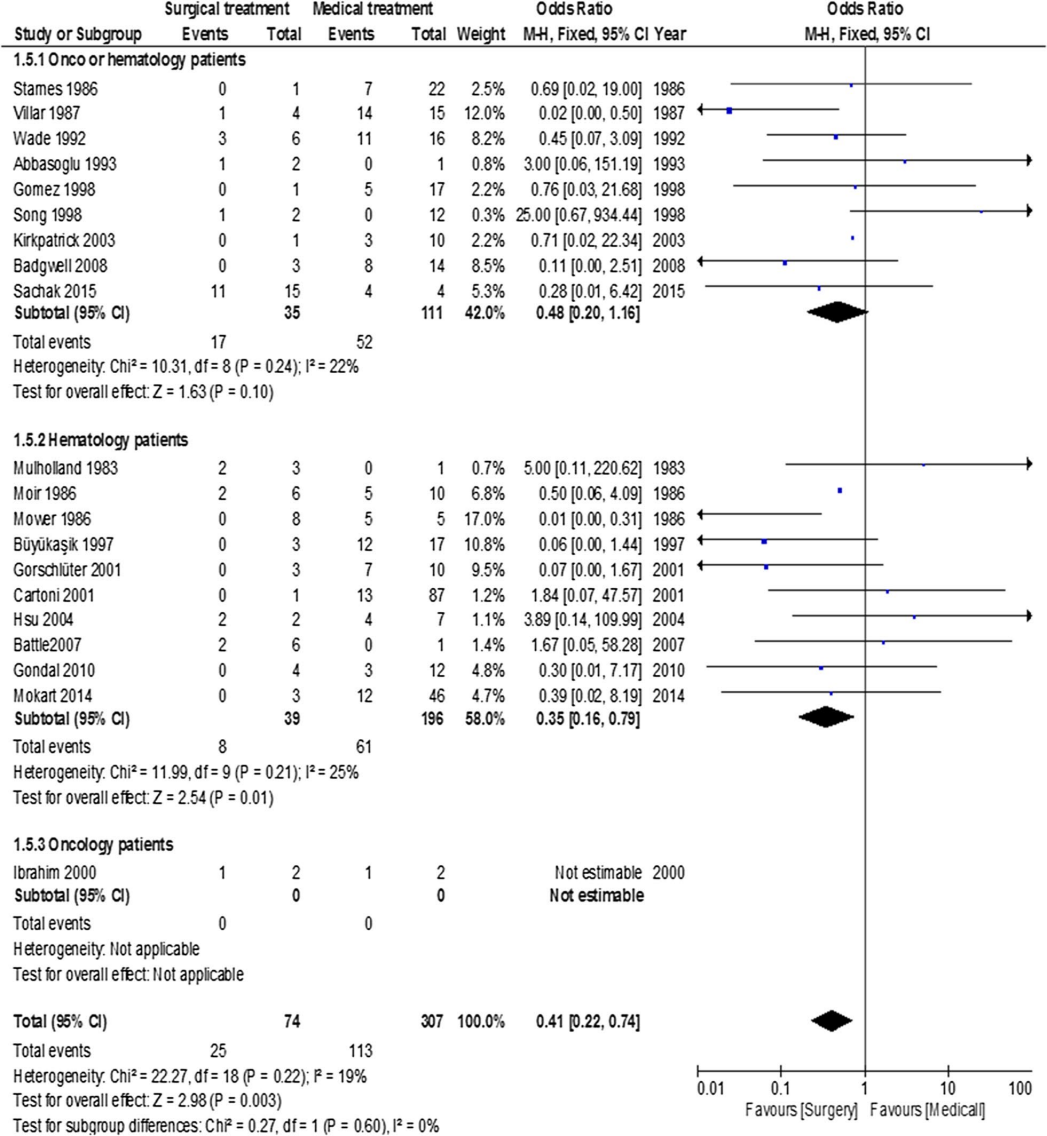
Summary of odds ratio in included studies according to underlying malignancy

In hematology patients, who usually undergo deeper and longer periods of neutropenia, surgery remains associated with a favorable outcome, suggesting that underlying malignancy did not influence outcome (Fig. 5). In studies with pooled oncology and hematology patients, patients undergoing surgery tended to have a better prognosis compared to patients receiving medical treatment, with an OR of 0.48 (95% CI = 0.2–1.16; *p* = 0.1). In studies focusing on patients with heamatological malignancies, the results of surgery were once again favorable with an OR of 0.35 (95% CI = 0.16–0.79; *p* = 0.01). The comparison between surgical and medical treatment could not be performed in oncology patients specifically, as only one publication focused on patients with solid tumors.

- Influence of neutropenia during the surgical procedure

**Fig. 6:**
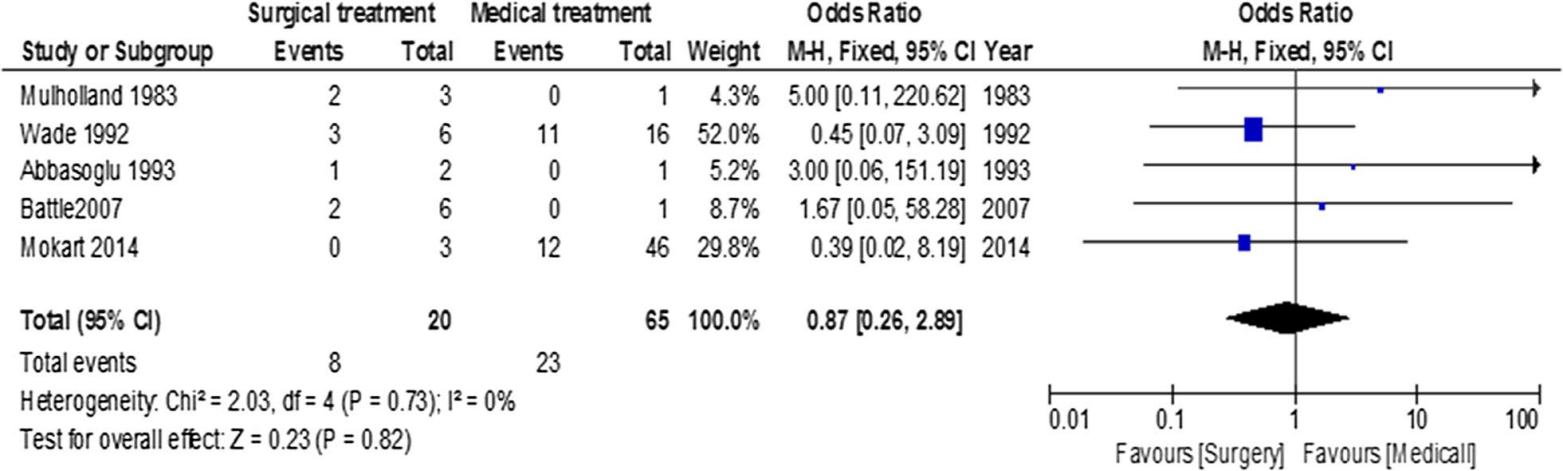
Summary of odds ratio in included studies according to the presence of neutropenia the day of surgery

Mortality according to the presence of neutropenia during the surgical procedure is displayed in Fig. 6. It assessed immediate surgery versus surgical procedures delayed after neutropenia resolution. The presence of neutropenia during surgical procedure, compared to patients medically treated, was not deleterious on outcome with an overall OR of 0.87 (95% CI = 0.26–2.89, *p* = 0.8).

## Discussion

This systematic review and meta-analysis, including 385 patients, assessed the prognostic association of abdominal surgery on outcome in neutropenic cancer patients presenting with NE compared to medical conservative treatment. It suggested that surgery was not associated with an increased mortality. According to our results, surgery was not deleterious, regardless of underlying malignancy, time period and the presence of neutropenia at the time of surgery. Interestingly, NE overall mortality was 42.2% (95% IC = 40.2–44.2), which is particularly encouraging compared to the literature from the 1980s. Moreover, recent data supported the good prognosis associated with NE in a large prospective study of critically ill neutropenic cancer patients admitted to the ICU [12].

The optimal management of NE has been a matter of debate [1, 8, 11, 51]. Physicians are frequently reluctant to select surgery as the first-choice treatment in neutropenic patients, based on a potential risk of higher infectious and hemorrhagic complications, although no publications support this idea. Interestingly, an appropriately early indication for appendectomy or cholecystectomy in neutropenic hematology patients was not associated with problematic postoperative course [52, 53]. Similarly, in 85 hematology patients who underwent surgery for acute abdominal complication, neutropenia and thrombopenia were not associated with outcome [54]. Moreover, data obtained in non-cancer patients with thrombocytopenia suggest that even high-risk hemorrhage surgical intervention such as splenectomy carried a low risk of morbidity and mortality [55].

Due to improvements in general supportive care, recent studies reported the success of conservative non-surgical management in most patients diagnosed with NE. It includes immediate broad-spectrum antimicrobial therapy adapted to local microbiological ecology and patients’ colonization [56–59], general supportive care (intravenous fluid support, parenteral nutrition and nasogastric suction if necessary, platelet transfusions in patients with severe thrombocytopenia, antalgic treatment) and bowel rest [8]. We could not analyze the impact of granulocyte colony stimulating factor (G-CSF) due to insufficient data. Its routine use remains of uncertain benefit and cannot be recommended [60]. Patients should be carefully monitored using repeated imaging to assess bowel wall thickness in addition to clinical response, as relapses can occur [61]. We found that the protective association of abdominal surgery with outcome tended to decrease over time compared to conservative treatment, probably because major advances have been made in the last decade in the medical management of severe sepsis and septic shock [19, 20], management of onco-hematology patients including in the ICU setting [21, 62] and including neutropenic patients [12, 22] and organ failures management [24–27]. Interestingly, surgery did not become deleterious, whereas medical management improved. Surgical interventions are generally reserved for selected cases of NE based on criteria first proposed by Shamberger et al., including: (a) the persistence of gastrointestinal bleeding despite correction of coagulopathy, thrombocytopenia and neutropenia; (b) free air in the intraperitoneal cavity indicative of bowel perforation; (c) clinical deterioration despite optimal medical management; and (d) the development of other indications for surgery such as appendicitis [63]. However, these criteria have never been evaluated. Another indication should be evaluated, concerning patients with bowel wall thickness greater than 10 mm, who carry a high mortality rate, because they may benefit from a surgical management [38].

Even when the surgery indication is clear, the optimal timing of surgery is debated. For symptomatic septic neutropenic patients, neutropenia recovery represents a high-risk period in which the clinical status is likely to worsen [64]. Waiting for neutropenia resolution remains debated because this approach might expose patients to a septic degradation toward septic shock. Interestingly, Badgwell recently suggested to delay surgery until neutropenia recovery, although he demonstrated in the same publication that surgery was independently associated with a good outcome, regardless of the duration of neutropenia, which appears as a conflicting message [11]. An expert panel from the French Intensive Care Society stated that neutropenia and thrombocytopenia should not modify the timing of surgery in patients with suspicion of digestive tract perforation [16], without any robust publication to rely on. Recent data demonstrated that preoperative septic shock and renal replacement therapy were independently associated with an increased mortality in hematology patients who underwent surgery for an acute abdominal complication [54]. We showed that surgery during the neutropenic period did not modify the prognosis, suggesting that surgery should probably not be delayed. It is important to note that some patients included in the meta-analysis underwent surgery lately at the stage of septic shock and multi-organ failures. Despite these severe situations, abdominal surgery was not associated with an increased mortality, suggesting that the prognostic impact of surgical management may be underestimated. We could not analyze early versus delayed surgical procedures. The influence of an early surgical strategy on outcome deserves to be evaluated, as we know that an early management is associated with a better prognosis [65, 66].

Our results indicated that surgery was not deleterious. Considering that inadequately treated typhlitis carries a high risk of death [6] and that the lack of surgical management was found to be a significant adverse prognostic factor [9, 11], larger indications of abdominal surgery should probably be evaluated. In tricky situations, exploratory laparotomy could probably be performed, as it seems not to be associated with an increased mortality, and represents an effective way to treat NE, perform microbiological samples and remove infectious inoculum. Pathological reports revealed that white laparotomy was uncommon. Infectious documentation is crucial in these patients, as the absence of diagnosis is a well-known adverse prognostic factor [27]. In the absence of microbial diagnosis, the place of empirical antifungal treatment is questionable, at the light of reported microbiological data.

We acknowledge several limitations. The main one is the strength of evidence in the literature concerning NE therapy, which is extremely poor. Available data are limited to low-quality studies, which are all retrospective, single-center, small-sample cohorts, case reports or case series. Moreover, there is a wide heterogeneity in patients, underlying malignancy, neutropenia duration and immunosuppression. There is also a bias in treatment allocation arm according to centers experience and case-volume, surgical indications differing among the studies. The wide admission period did not reflect all recent improvements and results can therefore be influenced. Moreover, study inclusion period was estimated using median inclusion period. This surrogate is, however, imperfect, a few studies being performed over large period. Lastly, several concerns existed with respect to the terminology of NE, because definition criteria evolved over time. It has been shown that clinical impressions are frequently inaccurate, initial clinical diagnosis being correct in only 53% of cases after autopsy or surgery confirmation [10]. Lastly, this study included various types of abdominal surgery, ranging from cholecystectomy to bowel necrosis with peritonitis, with different ranges of severity (no organ dysfunctions to multi-organ failure) prior to surgery, which can represent important cofounder factors.

However, in the absence of prospective studies or large retrospective cohorts, this meta-analysis may represent the best evidence supporting the absence of increased mortality associated with abdominal surgery in neutropenic cancer patients with NE. We do not know whether surgery is superior or comparable to medical treatment, but it did not appear as deleterious. However, surgical therapy can be useful. Delaying surgical therapy due to neutropenia, thrombocytopenia, or other chemotherapy or malignancies associated reasons is not recommended.

These data strengthen the indications of surgical management in the cases of GI or septic complications and question the place of surgery in other cases. These results may lead to conduct future clinical trials, including homogeneous cohorts of patients in terms of abdominal surgery and organ failure severity, in order to determine optimal surgery indications and evaluate the place of early surgical management in this context.

## Conclusions

NE is a diagnostic and therapeutic challenge associated with a high mortality rate, with controversial opinions on its optimal management. This systematic review and meta-analysis suggested the absence of excess risk of abdominal surgery on outcome versus conservative medical treatment in neutropenic cancer patients presenting with NE. Major advances have been made in the management of sepsis and supportive care in onco-hematology patients, making medical treatment essential in all cases. However, surgery appeared to be associated with a favorable outcome when indicated. Additional studies are needed to confirm these results and investigate the best indications of surgical treatment.

## Abbreviations

CI: confidence interval
GI: gastro-intestinal
ICU: intensive care unit
MOOSE: meta-analysis of observational studies in epidemiology
NE: neutropenic enterocolitis
OR: odds ratio
PRISMA: preferred reporting items for systematic reviews and meta-analyses
